# Generation of Humoral Immune Responses to Multi-Allele *Pf*AMA1 Vaccines; Effect of Adjuvant and Number of Component Alleles on the Breadth of Response

**DOI:** 10.1371/journal.pone.0015391

**Published:** 2010-11-03

**Authors:** Kwadwo A. Kusi, Bart W. Faber, Vanessa Riasat, Alan W. Thomas, Clemens H. M. Kocken, Edmond J. Remarque

**Affiliations:** 1 Department of Parasitology, Biomedical Primate Research Centre, Rijswijk, The Netherlands; 2 Department of Immunology, Noguchi Memorial Institute for Medical Research, College of Health Sciences, University of Ghana, Accra, Ghana; Federal University of São Paulo, Brazil

## Abstract

There is increasing interest in multi-allele vaccines to overcome strain-specificity against polymorphic vaccine targets such as Apical Membrane Antigen 1 (AMA1). These have been shown to induce broad inhibitory antibodies *in vitro* and formed the basis for the design of three *Di*versity-*Co*vering (DiCo) proteins with similar immunological effects. The antibodies produced are to epitopes that are shared between vaccine alleles and theoretically, increasing the number of component AMA1 alleles is expected to broaden the antibody response. A plateau effect could however impose a limit on the number of alleles needed to achieve the broadest specificity. Moreover, production cost and the vaccine formulation process would limit the number of component alleles. In this paper, we compare rabbit antibody responses elicited with multi-allele vaccines incorporating seven (three DiCos and four natural AMA1 alleles) and three (DiCo mix) antigens for gains in broadened specificity. We also investigate the effect of three adjuvant platforms on antigen specificity and antibody functionality. Our data confirms a broadened response after immunisation with DiCo mix in all three adjuvants. Higher antibody titres were elicited with either CoVaccine HT™ or Montanide ISA 51, resulting in similar *in vitro* inhibition (65–82%) of five out of six culture-adapted *P. falciparum* strains. The antigen binding specificities of elicited antibodies were also similar and independent of the adjuvant used or the number of vaccine component alleles. Thus neither the four extra antigens nor adjuvant had any observable benefits with respect to specificity broadening, although adjuvant choice influenced the absolute antibody levels and thus the extent of parasite inhibition. Our data confirms the feasibility and potential of multi-allele *Pf*AMA1 formulations, and highlights the need for adjuvants with improved antibody potentiation properties for AMA1-based vaccines.

## Introduction

Malaria is a disease caused by the apicomplexan parasite *Plasmodium* and remains a global scourge despite recent coordinated efforts at control and prevention in endemic areas. The development of an effective malaria vaccine has become ever more urgent in the face of increasing parasite/vector resistance to currently available drugs/insecticides for disease treatment and vector control [1]–[3]. Subunit vaccine development requires the identification of immunogenic targets from the wide array of antigens expressed by the parasite. A number of antigens expressed by *Plasmodium falciparum*, the parasite mostly responsible for severe forms of malaria, are currently at various stages of pre-clinical and clinical testing [4]–[6] with apical membrane antigen 1 (AMA1) being an important candidate. The vaccine properties and potential of AMA1 have recently been reviewed [7].

Despite the current global interest in AMA1 as a vaccine component, its potential is limited by allelic polymorphism resulting from single amino acid substitutions in an estimated 16% of amino acids (100 out of 622 positions in 1294 sequences accessed from GenBank) within the AMA1 molecule (Remarque, unpublished data). Polymorphism is generally an immune evasion strategy of the parasite that renders a fully functional immune response against one parasite strain less effective against strains expressing other allele variants, and this has been extensively demonstrated for AMA1 in murine models [8]–[10] as well as in clinical trials in malaria-naive populations [11]. This suggests that the immune response to AMA1, believed to be mainly antibody-mediated, is to both strain-specific and conserved epitopes [12]–[14]. We have previously shown that for any *Pf*AMA1 allele, the cross-strain anti-*Pf*AMA1 antibody fraction inhibits the homologous parasite strain *in vitro* to a similar extent as the total antibody fraction (strain-specific + cross-reactive) when both are tested at the same concentration [15]. On this premise, a universally effective *Pf*AMA1 vaccine should have two basic features; i) be able to induce functional responses to antibody epitopes that are common to, or conserved in the widest diversity of parasite strains possible, and ii) be such that strain-specific epitopes on the component alleles are diluted out or eliminated from the epitope repertoire. Attempts at achieving such responses have been made through immunisation with recombinant vaccines incorporating a number of *Pf*AMA1 alleles [15]–[17], and have been the basis for the *in silico* design of three *Di*versity-*Co*vering (DiCo) *Pf*AMA1-based proteins with similar immunological effects when mixed in a vaccine formulation [18]. The three DiCo proteins, incorporating 80–97% of AMA1 amino acid diversity found naturally at the time of design, are distinctly different from one another and are together expected to elicit functional cross-strain antibodies. Initial functional assays with protein A-purified rabbit antibodies (6 mg/ml) against DiCo mix in Montanide ISA 51 showed an average *in vitro* inhibition of 70% against the highly diverse FVO, HB3 and 3D7 parasite strains [18].

It seems reasonable to assume that increasing the number of different *Pf*AMA1 alleles included in such a multi-allele vaccine would further broaden the specificity of the expected anti-*Pf*AMA1 antibody response. This idea raises the question of how many alleles would be necessary to achieve the broadest possible functional antibody response. It should also be noted that the number of alleles that can be included in such a vaccine would be limited by the cost of producing many different antigens prior to mixing, and the potential inherent difficulties with quality assurance/control required for vaccine formulation. Another level of complexity would be the appropriate adjuvant system for vaccine formulation. Adjuvants are known to modulate the specificity of antibodies elicited against some parasite antigens [19], [20].

In the present study, we further examine the broadened antibody response after immunisation of rabbits with a mixture of the three DiCo proteins by competition ELISA and *in vitro* growth inhibition assays (GIAs) with six distinct *P. falciparum* parasite strains. We compare this response with that induced by a similar immunisation with a mixture of seven antigens (the three DiCo proteins and natural AMA1 alleles from FVO, HB3, 3D7 and CAMP strains of *P. falciparum*) for gains in broadened specificity. We also assess the effect of the choice of adjuvant on the antigen specificities of antibodies elicited against the DiCo mix vaccine.

Our data suggests a practical limit to the number of *Pf*AMA1 alleles that need to be incorporated in a multi-allele vaccine to achieve broad specificity, and shows no adjuvant effect on antigen binding specificity of the elicited antibodies. It also highlights a potentially important role for cross-reactive antibodies in naturally acquired immunity to malaria, and the need for new adjuvants with improved antibody potentiation properties and safety for use in *Pf*AMA1-based subunit vaccine development.

## Methods

### Ethics Statement

All animals used in this study were handled in strict accordance with good animal practices within the respective jurisdictions (German and European Union guidelines for BioGenes GmbH, Berlin, and the Belgian national animal welfare regulations for Eurogentec SA, Seraing). Rabbit immunisation work at BioGenes was under approval from NIH/OLAW (ID number #A5755-01) and immunisations at Eurogentec had approval from the ethics committee of the Centre d'Economie Rurale (CER Groupe, Marloie, Belgium).

### Protein Production, Adjuvants and Rabbit Immunisations

The full ectodomain of the AMA1 allelic forms from *P. falciparum* strains FVO, HB3, 3D7 and CAMP, as well as the *in silico*-designed DiCo 1, DiCo 2 and DiCo 3 antigens were expressed as recombinant proteins in *Pichia pastoris* by a similar methodology as described elsewhere [21]. Groups of rabbits were immunised with *Pf*AMA1 multi-allele formulations in three different adjuvants. CoVaccine HT™, an enhancer of both humoral and cellular immune responses, is a novel proprietary vaccine adjuvant from Protherics Medicines Development Limited, a BTG Company (London, UK). It is a squalene-based oil-in-water adjuvant that has a sucrose fatty acid sulphate ester (SFASE) [22]. Montanide ISA 51 is a water-in-oil emulsion developed by SEPPIC (Paris, France), containing the naturally metabolizable oil Drakeol 6 VR and mannide mono-oleate as emulsifier, and the adjuvant promotes antibody formation and significant cytotoxic T-cell activity [23], [24]. Montanide IMS 4112 VG PR, (one of the Montanide IMS group of adjuvants from SEPPIC), is a water-dispersible composition containing (undisclosed) immuno-stimulatory organic compounds and excipient [25].

The first group of rabbits (Gp 1, n = 9) was immunised with a mixture of 7 antigens (DiCo 1, DiCo 2, DiCo 3, and *Pf*AMA1 allelic antigens from FVO, HB3, 3D7 and CAMP parasite strains, ∼7 µg of each antigen, 50 µg total antigen per dose) in CoVaccine HT™. The second group (Gp 2, n = 8) was immunised with a mixture of 3 antigens (∼17 µg each of DiCo 1, DiCo 2 and DiCo 3, here referred to as DiCo mix; 50 µg in total) in CoVaccine HT™. The third group of rabbits (Gp 3, n = 8) was immunised with DiCo mix (∼17 µg of each DiCo protein, 50 µg in total) in Montanide IMS, and the fourth group (Gp 4, n = 5) with DiCo mix (10 µg of each DiCo protein, 30 µg in total) in Montanide ISA 51. All immunisations were done intramuscularly with 3 full human doses (500 µl/dose) of vaccine on days 0, 28 and 56, and exsanguination for all groups was on day 70. Vaccine formulation in all cases was according to the adjuvant manufacturers' specifications. For CoVaccine HT™, formulation involved mixing 300 µl of antigen at 200 µg/ml with 300 µl of CoVaccine HT™ with an SFASE concentration of 40 mg/ml. One 500-µl dose of the resulting formulation therefore contained 50 µg antigen and 10 mg SFASE. Montanide IMS formulation also involved mixing 300 µl of antigen with 300 µl of adjuvant such that 500 µl of the resulting mixture contained 50 µg of antigen. Formulation with Montanide ISA 51 was in a 50/50 (w/w) ratio of antigen to adjuvant; 276 µl of antigen at 130 µg/ml was mixed with 324 µl of adjuvant and the mixture was emulsified by 20 passages through a 22-gauge syringe-coupling piece prior to injection with 500 µl.

Rabbits in the first three immunisation groups were housed at BioGenes GmbH (Berlin, Germany), while the last group (Gp 4) was housed at Eurogentec SA (Seraing, Belgium). The second and third group immunisations (Gp 2 and Gp 3) were done simultaneously in the same experiment at BioGenes, while the first and fourth (Gp 1 and Gp 4) were done in separate experiments. Final bleed sera from the four different immunisation groups were used in the assays described here.

### Antibody Purification

Antibodies from all groups were purified on Protein G sepharose (GE Healthcare, Etten-Leur, The Netherlands) columns. Binding and elution buffers (Pierce, Rockford, IL) were used according to manufacturer's protocols. After elution, antibody eluates were filtered (0.22 µm), concentrated and exchanged into RPMI 1640 using pre-sterilized AmicronUltra-15 tubes (30-kDa cutoff; Millipore, Ireland). The concentration of each antibody fraction was subsequently determined with a Nanodrop ND1000 spectrophotometer using the IgG molar extinction coefficient (Nanodrop Technologies, Wilmington, DE) and stored at −20°C until use.

### ELISA

Competition ELISA was performed as previously described [15]. Briefly, 96-well flat bottom Microlon titre plates (Greiner, Alphen a/d Rijn, The Netherlands) were coated with 1 µg/ml (100 µl/well) of the relevant antigen (DiCo 1, DiCo 2, DiCo 3, FVO AMA1, HB3 AMA1, CAMP AMA1 or 3D7 AMA1) at 4°C overnight. After blocking with 200 µl/well of 3% BSA in PBS-T, 60 µg/ml of each of six competitor/soluble antigens (AMA1 from strains FVO, HB3, 3D7 or CAMP, a mixture of the 4 AMA1 alleles designated NM, and DiCo mix designated DM) were titrated 3-fold over 9 duplicate wells at a 50 µl/well final volume. Fifty microlitres/well of a fixed dilution (two arbitrary units or 2 AU) of rabbit IgGs (titre pre-determined using the relevant capture antigen) was then added to the titrated competitor antigens and co-incubated for 2 h. A pool of sera from rabbits immunised with a mixture of 3 *Pf*AMA1 alleles (FVO, HB3, 3D7 strains), titrated 2-fold from 1∶100,000, was used as standard calibrator on all plates. After sample incubation, plates were developed with 100 µl/well of 1∶1250-diluted goat anti-rabbit IgG/alkaline phosphatase conjugate (Pierce, Rockford, IL). Colour development was with 100 µl/well of p-nitrophenyl phosphate (pNPP; Fluka, Poole, UK) for 30 min. and the optical density (OD) at 405 nm determined.

Measured ODs were converted to arbitrary units (AUs) by the standard curve included on each plate and expressed as a percentage of AU values from wells without competitor antigen. Residual binding (%) at the highest competitor antigen concentration was estimated from AU values based on a least squares approximation from the following four-parameter logistic function;



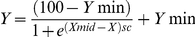
where *Y* is the predicted % residual binding, ***Y_min_*** is the maximal depletion at infinite soluble antigen concentration (minimum value), ***X*** is the soluble antigen concentration (log scale), ***X_mid_*** is the soluble antigen concentration (log scale) at which 50% antibody depletion is achieved (midpoint between the maximum and minimum depletion values), and ***sc*** is the slope of the curve. Percent antibody depletion for any competitor/soluble antigen is therefore the difference between 100% (binding in the absence of soluble antigen) and residual binding at the highest competitor antigen concentration of 30 µg/ml.

### Parasite Cultures and Growth Inhibition Assays

Protein G-purified IgG fractions were tested for *in vitro* activity in parasite growth inhibition assays (GIAs). All IgGs were tested in triplicate on FCR3 (one amino acid difference in the pro-domain from the FVO strain, with *ama1* GenBank accession no. M34553), NF54 (parent strain of the 3D7 clone with *ama1* GenBank accession no. U65407), HB3 (accession no. U33277), L32 (accession no. EF221749), 7G8 (accession no. M34555) and CAMP (accession no. M34552) parasite strains at a 2-fold serial dilution from 6 mg/ml in 96-well half area cell culture plates (Greiner, Alphen a/d Rijn, The Netherlands). Parasites were cultured under standard conditions (an atmosphere of 5% CO_2_, 5% O_2_, and 90% N_2_, 37°C), and the *Pf*AMA1 antigens expressed by all parasite strains were verified by PCR and restriction fragment length analysis. Parasite cultures were mycoplasma-free and synchronized with 0.3 M Alanine, 10 mM Hepes pH 7.5 before use in assays. Late trophozoite/early schizont stages at a parasitaemia of 0.3±0.1% and 2% final haematocrit were used in all assays. The final culture volume was 50 µl/well and parasites were incubated for 42–46 h. Parasite growth was assessed by measuring parasite lactate dehydrogenase levels and plates were read at 655 nm after 30 min of development. Parasite growth inhibition was expressed as;



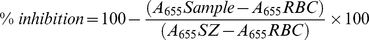
where *A_655_Sample* is the OD_655_ for any test sample well, *A_655_SZ* is the average OD_655_ of schizont control wells included on each plate and *A_655_RBC* is the average OD_655_ of RBC control wells. The data is presented as the arithmetic mean % inhibition from each sample triplicate.

### Statistical Analyses

Residual antibody binding (***Y_min_***) for each competitor antigen in competition ELISA was estimated by a 4-parameter logistic fit with least squares approximation using the *R* statistical package (R Development Core Team, 2009, version 2.10.1). The mean % depletion (100-***Y_min_***) and 95% confidence intervals of a competitor antigen on one capture antigen were calculated and compared with mean values of the same competitor antigen on other capture antigens within the same immunisation group, or with mean values obtained for the same competitor antigen in other immunisation groups.

ELISA antibody titres on seven different capture antigens are presented as dotplots superposed with boxplots indicating the median as well as the lower and upper quartiles for each immunisation group. GIA data is presented as the mean % growth inhibition ± standard error of mean per immunisation group against each of the six parasite strains tested. Associations between antibody titre and GIA activity for four parasite strains with matching ELISA data were estimated with a four-parameter logistic fit.

The original Gp 1 study involved 98 rabbits immunised with the seven-antigen mixture in CoVaccine HT™. ELISA was performed on a random sample of 9 out of the 98 available. Since small volumes (∼100 µL) of the individual rabbit sera were available, only a single antibody sample, representing a pool of IgGs from the 98 rabbits, was available for *in vitro* growth inhibition assay. The growth inhibitory capacity of this single sample against three parasite strains (FCR3, HB3, NF54) was therefore compared directly with that of IgGs purified from a pool of sera from all 8 rabbits immunised with DiCo mix in CoVaccine HT™ (Gp 2).

All plots were prepared with the *R* statistical package.

## Results

### Three-Antigen and Seven-Antigen Immunisations Induce Antibodies with Similar Specificity Profiles

**Figure 1:**
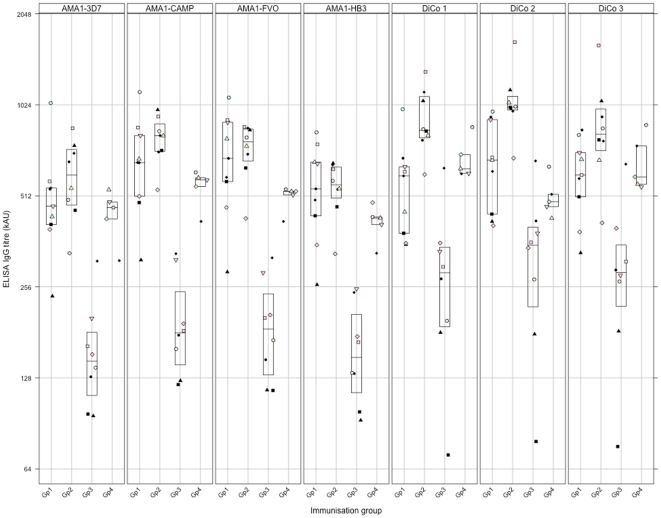
Levels of anti-AMA1 antibody elicited with the four multi-allele vaccine formulations in rabbits. Gp 1 rabbits were immunised with seven AMA1 antigens (DiCo mix and AMA1 from FVO, HB3, 3D7 and CAMP parasite strains) in CoVaccine HT™, and the other groups were immunised with DiCo mix in CoVaccine HT™ (Gp 2), Montanide IMS (Gp 3) and Montanide ISA 51 (Gp 4) respectively. Antibody titres were determined by a standardized ELISA with DiCo 1, DiCo 2, DiCo 3, FVO, HB3, 3D7 and CAMP AMA1-coated plates. Data is presented on a Log2 scale as dotplots with a boxplot superpose indicating the lower and upper quartiles as well as the median per immunisation group. Within the same immunisation group, plotting symbols represent the antibody titre of individual rabbits on all coating/capture antigens.

Specificity profiles of antibodies from rabbit immunisations with the three-antigen (DiCo mix, Gp 2) and seven-antigen (DiCo mix + AMA1 alleles from FVO, HB3, 3D7 and CAMP strains of *P. falciparum*, Gp 1) vaccines, both formulated in CoVaccine HT™, were determined by a standardized competition ELISA with the three DiCo proteins used separately as capture antigens. Initial titrations with the separate DiCos as capture antigens showed that antibody titres were higher (1.4–1.7 times) in the three-antigen immunisation group (Gp 2) compared to the seven-antigen immunisation group (Gp 1), and the differences were statistically significant when DiCo1 and DiCo 2 were used as capture antigens (Figure 1).

In competition ELISA, antibodies from the three and seven-antigen immunisation regimens were co-incubated with four natural *Pf*AMA1 alleles (from 3D7, HB3, FVO and CAMP parasite strains) and two different antigen mixtures (DiCo mix or DM, and a mixture of the four natural *Pf*AMA1 alleles, designated NM) in coated and blocked plates. Antibody depletion by all 6 competitor antigens/mixtures was fairly consistent for all rabbits within the same immunisation group when assays were done on DiCo 1, DiCo 2 and DiCo 3-coated plates, respectively. Statistical comparison of all rabbit antibodies from the three-antigen (Gp 2, n = 8) and seven-antigen (Gp 1, n = 9) immunisation groups, both formulated in Co-Vaccine HT, showed no significant difference in mean % antibody depletion by all six competitor antigens/mixtures (Table 1, Gps 1 & 2). For both vaccines, antibody depletion by the competitor antigen mixtures (DM and NM) was between 91% and 99% on all three DiCo capture antigens as was expected (Table 1). Of the four natural *Pf*AMA1 competitor antigens, HB3 AMA1 depleted the most antibodies (87–95%) on all the DiCo capture antigens, whilst 3D7 AMA1 depleted the least (67–82%) (Table 1, Gps 1 & 2). The mean % antibody depletion amongst competitor antigens varied most on DiCo 2-coated plates compared to DiCo 1- and DiCo 3-coated plates. Mean % antibody depletion was least for 3D7 AMA1 (∼68%) on DiCo 2-coated plates, irrespective of whether the antibodies were raised with the three- or seven-antigen vaccines. By contrast, mean % antibody depletion was greatest for FVO AMA1 competitor antigen (86.4% and 90.0% respectively for antibodies raised in the three- and seven-antigen immunisations) and HB3 AMA1 (94.4% and 94.0%, respectively) on DiCo 2-coated plates. 3D7 AMA1 as a competitor antigen depleted most antibodies from both immunisation groups (80.6% and 82.1% respectively for antibodies raised in the three- and seven-antigen immunisations) on DiCo 3-coated plates (Table 1, Gps 1 & 2).

**Table 1:** Mean % antibody depletion from DiCo 1, 2 and 3-coated plates.

Capture antigen	Competitor antigen	Gp 1 (7Ag/HT) n = 9	Gp 2 (3Ag/HT) n = 8	Gp 3 (3Ag/IMS) n = 8	Gp 4 (3Ag/ISA 51) n = 5
DiCo 1	FVO	84.7(81.6–87.8)	83.0(79.5–86.6)	77.0(71.9–82.0)	77.4(73.0–81.8)
	HB3	93.9(91.7–96.1)	90.1(88.5–91.6)	90.7(87.9–93.6)	83.4(76.7–90.1)
	DM	96.1(93.9–98.3)	98.6(98.2–99.1)	97.2(95.9–98.4)	91.9(88.3–95.5)
	CAMP	87.6(84.2–90.9)	86.3(84.5–88.0)	78.3(74.5–82.1)	77.4(71.4–83.5)
	3D7	75.2(72.2–78.2)	76.9(73.1–80.8)	69.3(65.0–73.6)	61.4(54.9–68.0)
	NM	97.6(96.1–99.0)	92.4(91.3–93.4)	92.1(90.8–93.5)	88.2(85.8–90.6)
DiCo 2	FVO	90.0(87.2–92.8)	86.4(84.2–88.6)	84.8(79.4–90.2)	83.6(81.2–86.1)
	HB3	94.0(91.8–96.2)	94.4(91.1–97.8)	97.1(94.8–99.4)	83.7(71.6–95.7)
	DM	96.1(93.9–98.4)	97.6(96.4–98.9)	98.1(96.6–99.6)	92.9(90.4–95.5)
	CAMP	83.0(79.6–86.4)	83.5(80.1–87.0)	77.9(75.3–80.5)	77.3(76.1–78.6)
	3D7	67.6(63.3–71.9)	68.3(65.9–70.6)	63.6(58.7–68.5)	56.6(48.3–64.9)
	NM	95.0(93.7–96.4)	92.9(91.2–94.7)	95.0(92.2–97.8)	88.9(85.5–92.3)
DiCo 3	FVO	81.2(77.7–84.6)	81.9(78.2–85.6)	72.8(66.9–78.7)	74.1(66.8–81.4)
	HB3	91.9(89.6–94.3)	87.1(85.2–88.9)	85.1(82.5–87.7)	80.9(74.8–87.0)
	DM	97.3(92.2–102.2)	97.8(96.4–99.3)	96.8(95.4–98.2)	91.0(89.1–92.9)
	CAMP	80.2(77.0–83.3)	80.8(76.7–84.9)	66.3(59.7–73.0)	67.8(62.5–73.1)
	3D7	82.1(78.6–85.6)	80.6(77.9–83.2)	66.5(61.4–71.7)	57.8(47.9–67.6)
	NM	94.2(93.1–95.4)	91.4(89.0–93.7)	86.5(83.6–89.4)	84.0(77.6–90.4)

Antibody depletion after competition ELISA, values reported as mean % depletion (95%CI).

7Ag – vaccine containing DiCo mix + four AMA1 alleles from FVO, HB3, 3D7 and CAMP parasite strains.

3Ag – vaccine containing DiCo mix.

NM &DM – competitor antigen mixtures comprising natural AMA1 alleles (FVO, HB3, 3D7, CAMP) and DiCo mix, respectively.

The 9 antibody samples from the seven-antigen immunisation in CoVaccine HT™ were randomly selected from a total of 98 such rabbits, and a pool of antibodies from all 98 rabbits was subsequently tested in GIA. The ELISA antibody depletion pattern of this IgG pool was shown to be similar to that of the individual antibody samples (data not shown).

**Figure 2:**
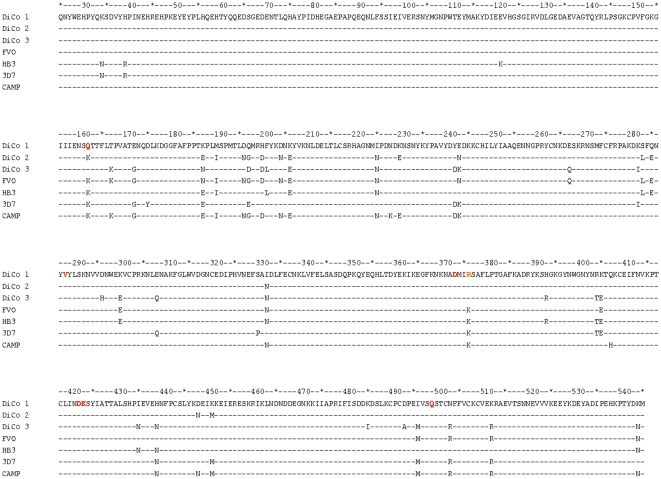
Alignment of the protein sequences (aa25-545) of *Pf*AMA1 antigens used in this study. DiCo proteins were used to immunise rabbits and as capture antigens in ELISA. Natural allele AMA1 proteins were used to immunize rabbits, and as capture and competitor antigens in (competition) ELISAs. All proteins were produced in *Pichia pastoris* and are devoid of N-glycosylation sites. These have been replaced with amino acid residues (indicated in red) that occur in AMA1 sequences from other malarial species (N162Q, T288V, S373D, N422D, S423K, N499Q). Residue 162 is unique as it is also a polymorphic residue. Additionally, all sequences contain a point mutation at position 376 (K to R, indicated in orange). This was necessary to prevent protein cleavage by *P. pastoris* proteases.

Differences in % antibody depletion amongst the four allelic *Pf*AMA1 competitor antigens may be related to the DiCo protein coverage of polymorphism found in natural alleles. Alignments of DiCo protein amino acid sequences with those of the four natural alleles (Figure 2) reveal that apart from position K376 which was mutated in the three DiCo proteins to avoid protein cleavage, there are five other positions in 3D7 AMA1 that are not present in any of the DiCo proteins. CAMP AMA1 sequences have two uncovered positions, HB3 AMA1 has three and FVO AMA1 has all amino acid residues represented at least once in the three DiCo proteins.

### The Majority of Antibodies Raised in Three-Antigen and Seven-Antigen Immunisations Recognise Shared Epitopes in Natural *Pf*AMA1 Alleles

In order to further test the hypothesis that multi-allele vaccines induce high proportions of antibodies to epitopes that are shared by the vaccine component alleles, the specificities of antibodies elicited by all four vaccine formulations to four different *Pf*AMA1 alleles were assessed by competition ELISA. Antibody titres were initially determined by a standard ELISA on plates coated with *Pf*AMA1 from FVO, HB3, 3D7 and CAMP parasite strains respectively, and titres were comparable to those obtained when the same antibodies were titrated on the immunising DiCo antigens (Figure 1). In our hands, rabbit immunisations with antigen from concentrations of 30–100 µg/ml have usually yielded similar results, hence the differences in the antigen dose between the first three groups and the DiCo mix/Montanide ISA 51 group (Gp 4) may not result in different titres. The four *Pf*AMA1 alleles (from FVO, HB3, 3D7 and CAMP parasite strains) were used as competitor antigens in subsequent competition ELISA on plates coated with FVO and 3D7 AMA1 antigens respectively. In assays performed with FVO AMA1-coated plates, antibodies from all four immunisation groups showed the greatest depletion with the homologous FVO AMA1 (94–98%) and HB3 AMA1 (94–97%) as expected (Table 2). CAMP AMA1 and 3D7 AMA1 depleted 78–84% and 67–82%, respectively. Likewise, assays performed with 3D7 AMA1-coated plates using the same rabbit antibodies resulted in 92–98% depletion by 3D7 AMA1, 85–86.5% depletion by CAMP AMA1, 88–90.5% depletion by HB3 AMA1 and 81–84% depletion by FVO AMA1 (Table 2). Between groups, antibody depletion by an individual competitor antigen was similar on the same capture antigen, and irrespective of the number of immunising antigens (Table 2, Gps 1 & 2) or the adjuvant used in vaccine formulation (Table 2, Gps 2, 3 & 4). On average, for all immunisation groups, the extent of recognition of FVO-binding antibodies by 3D7 AMA1 competitor antigen was about 8.8% less than the recognition of 3D7 AMA1-binding antibodies by FVO AMA1 competitor antigen. These observations indicate that though the majority of antibodies recognise shared epitopes in the four natural *Pf*AMA1 alleles, a fraction of antibodies are still strain-specific. This may be explained by assuming that some antigen-specific epitopes on any of the three DiCo proteins is/are not present on the *Pf*AMA1 alleles of interest. Antibodies elicited against such epitopes only recognize the *Pf*AMA1 allele(s) that have these epitopes and will not bind to alleles without these strain-specific epitopes. Additionally, local distortions of the protein structure resulting from changes in amino acids that are in close proximity to, or within these epitopes may mean antibodies will have lower binding avidity/affinity for these epitopes. This may result in a continuum of binding specificities, such that some antibodies will have high avidity for epitopes on some *Pf*AMA1 alleles and lower avidity for corresponding, albeit altered epitopes on other alleles. With the exception of FVO AMA1, the three other natural *Pf*AMA1 alleles have amino acids that are not present in any of the three DiCo proteins (Figure 2). Epitopes that include these polymorphic amino acids are therefore prime candidates that may explain the remnant strain-specificity observed.

**Table 2:** Mean % antibody depletion from FVO and 3D7 AMA1-coated plates.

Capture antigen	Competitor antigen	Gp 1 (7Ag/HT) n = 9	Gp 2 (3Ag/HT) n = 8	Gp 3 (3Ag/IMS) n = 8	Gp 4 (3Ag/ISA 51) n = 5
3D7 AMA1	FVO	83.9(81.6–86.2)	85.4(81.8–89.0)	81.4(78.1–84.7)	82.8(79.1–86.5)
	HB3	90.5(89.2–91.8)	89.8(88.4–91.1)	90.5(88.0–93.0)	88.1(85.3–91.0)
	CAMP	86.4(84.1–88.7)	86.5(81.9–91.2)	85.6(81.7–89.4)	85.3(82.6–88.0)
	3D7	96.8(95.7–97.9)	98.1(96.6–99.6)	98.0(95.3–100.7)	92.5(90.3–94.7)
FVO AMA1	FVO	96.1(95.3–96.8)	97.5(96.3–98.7)	97.5(96.4–98.6)	94.3(93.0–95.6)
	HB3	95.0(93.9–96.0)	95.7(94.5–96.8)	96.3(95.5–97.1)	94.1(92.7–95.6)
	CAMP	82.9(80.6–85.2)	83.6(80.5–86.6)	78.3(74.9–81.7)	83.6(79.5–87.7)
	3D7	73.4(70.5–76.3)	81.8(79.7–83.9)	75.5(70.2–80.8)	67.7(61.8–73.7)

Antibody depletion after competition ELISA, values reported as mean % depletion (95%CI).

7Ag – vaccine containingDiCo mix + four AMA1 alleles from FVO, HB3, 3D7 and CAMP parasite strains.

3Ag – vaccine containing DiCo mix.

NM &DM – competitor antigen mixtures comprising natural AMA1 alleles (FVO, HB3, 3D7, CAMP) and DiCo mix, respectively.

### The Choice of Adjuvant Determines the Quantity but not the Antigen Specificity of Elicited Antibodies

In order to assess the effect of adjuvant on the specificity of elicited responses, rabbit antibodies raised against DiCo mix in Co-Vaccine HT (Gp 2, n = 8) were compared with those raised in separate immunisations with DiCo mix in Montanide IMS (Gp 3, n = 8) and Montanide ISA 51 (Gp 4, n = 5) as adjuvants. Antibody titres were dependent on the vaccine adjuvant when titrated in DiCo 1-, DiCo 2- or DiCo 3-coated plates. Antibody titres were generally comparable in the immunisation groups with Co-Vaccine HT and Montanide ISA 51, whilst titres were comparatively lower in the Montanide IMS group (Figure 1, Gps 2, 3 & 4)). Additionally, depletion patterns in competition ELISA showed a general, albeit insignificant trend of higher % depletions of antibodies against DiCo mix in CoVaccine HT™ (Gp 2) by all competitor antigens/mixtures, with 3D7 and CAMP AMA1 competitor antigens showing only borderline significance in some instances (Table 1, Gps 2, 3 and 4). Depletion of antibodies from the two Montanide immunisation groups was also generally comparable except for the two competitor antigen mixtures DM and NM. Thus though the choice of an adjuvant for immunisation affected the physiological levels of anti-DiCo mix antibodies in rabbits, the current data suggests that the adjuvant effect on epitope presentation to B cells, and by extension on the antibody specificity, may only be marginal. This may however be applicable only to the three adjuvants used in this study.

### *In Vitro* Functional Assays with Anti-DiCo Mix Antibodies Show Similar Inhibition of Multiple *P. falciparum* Strains

The functional activity of anti-DiCo mix antibodies from the four immunisation groups was determined *in vitro* on a broad panel of culture-adapted *P. falciparum* strains (FCR3, NF54, HB3, L32, 7G8 and CAMP). Antibodies were tested at a 2-fold dilution from 6–0.75 mg/ml against all parasite strains.

**Figure 3:**
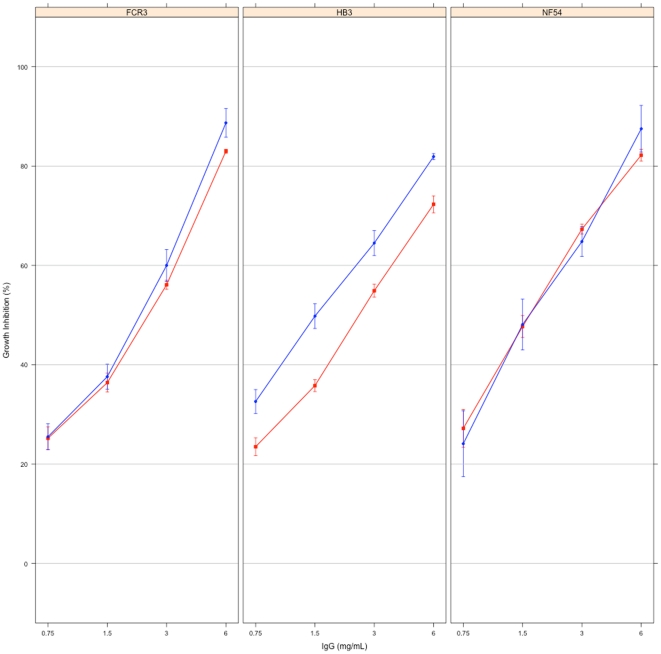
Growth inhibition of *Plasmodium* parasites by antibody pools from the 3-antigen and 7-antigen immunisation groups. The 7-antigen immunisation group (Gp 1) had only a single sample (antibodies purified from a pool of 98 rabbit sera), hence a direct comparison of the functional activities of antibodies from the 3 and 7-antigen immunisation groups could not be made. The growth inhibitory activity of IgGs purified from a pool of all rabbits immunised with DiCo mix (3-antigen, Gp 2) in the same adjuvant (CoVaccine HT™) was therefore compared with that of the single Gp 1 sample against the FCR3, HB3 and NF54 parasite strains. Plots represent the mean % inhibition ± SEM for replicate measurements for each sample. Blue filled circles (•) represent the Gp 1 pooled sample and the red filled squares (▪) represent the Gp 2 pooled sample.

**Figure 4:**
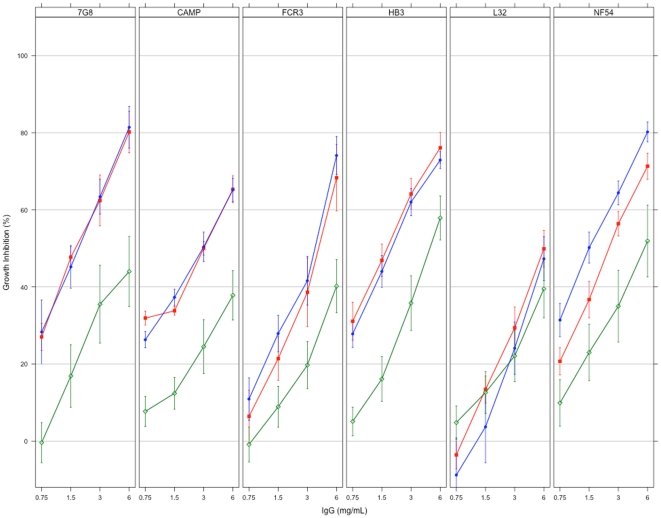
Growth inhibition of *Plasmodium* parasites by antibodies elicited with the three DiCo mix vaccines. Antibodies from all immunisation groups were tested on each of six culture-adapted strains (7G8, CAMP, FCR3, HB3, L32 and NF54) of *P. falciparum*. Plots represent the mean % inhibition ± SEM for all antibody samples within an immunisation group. Blue filled circles (•) represent Gp 2 (DiCo mix in CoVaccine HT™, n = 8), green open diamonds (◊) represent Gp 3 (DiCo mix in Montanide IMS, n = 8) and red filled squares (▪) represent Gp 4 (DiCo mix in Montanide ISA 51, n = 5).

At the highest concentration tested, antibodies from the seven-antigen immunisation in CoVaccine HT™ (Gp 1, n = 1, representing a pool with n = 98) showed % growth inhibition of 75.1%, 81.9%, 87.2%, 88.3%, 89.1% and 93.9% against the L32, HB3, NF54, 7G8, FCR3 and CAMP parasite strains, respectively. The single sample available for testing in this group however meant these values could not be directly compared with the mean % inhibition of the 5 or 8 different rabbit IgGs in the other immunisation groups. The functional activity of this sample against three of the six parasite strains (FCR3, HB3, NF54) was therefore compared with that of IgGs purified from a serum pool from all 8 rabbits immunised with DiCo mix in CoVaccine HT™ (Gp 2) in separate experiments. The % inhibition of pooled antibodies from the seven-antigen immunisation were the same as that of the DiCo mix pooled antibodies against NF54 and FCR3 parasite strains, but slightly higher than that of DiCo mix pooled antibodies against HB3 parasites (Figure 3). Moreover, the % inhibition of these three parasite strains by the DiCo mix pooled antibodies compared favourably with the corresponding mean % inhibition estimated from the measured inhibitions of the individual rabbit IgGs to DiCo mix in CoVaccine HT™ (Figures 3 and 4).

**Figure 5:**
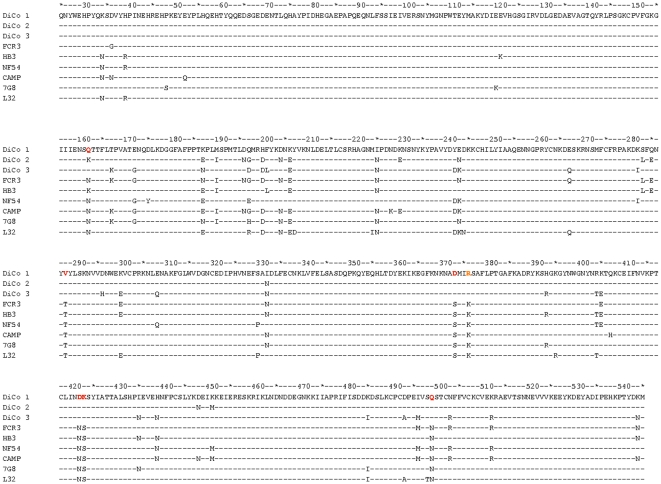
Alignment of DiCo protein sequences (aa25-545) with those of AMA1 present in GIA malaria parasites. AMA1 protein sequences of malaria parasites used for *in vitro* growth inhibition assays were accessed from the GenBank database. The DiCo sequences contain point mutations at the cleavage (K376R, indicated in orange) and potential N-glycosylation (N162Q, T288V, S373D, N422D, S423K, N499Q) sites (indicated in red), and differ from parasite AMA1 sequences at these sites. Amino acids at 51 polymorphic sites (within aa25-525) also differ between sequences.

The level of *in vitro* inhibition was the same for all the three DiCo mix immunisation groups (Gp 2, Gp 3 and Gp 4) against five of the six parasite strains, with the L32 strain being the exception (Figure 4). At the highest concentration tested (6 mg/ml), mean % growth inhibition of antibodies induced against DiCo mix in CoVaccine HT™ (Gp 2, n = 8) was between 65 and 82% whilst that of antibodies against DiCo mix in Montanide ISA 51 (Gp 4, n = 5) was between 65 and 81% against the five strains (Figure 4). Antibodies against DiCo mix in Montanide IMS (Gp 3, n = 8) had the lowest mean % growth inhibitions (between 37% and 58%) against all five strains (Figure 4). Although inhibition of the L32 strain was generally lower in all immunisation groups (47.3% for Gp 2, 39.5% for Gp 3 and up to 50% for Gp 4 at 6 mg/ml total IgG), the trend across immunisation groups was similar to that for the other parasite strains. The observed lower inhibition of L32 parasites may be explained by the many polymorphic amino acids (R197, D207, I224, N244, P330, R395 and T498) within the L32 AMA1 sequence that do not occur in any of the three DiCo proteins, aside those in the prodomain, and mutations at N-glycosylation and cleavage sites (Figure 5).

**Figure 6:**
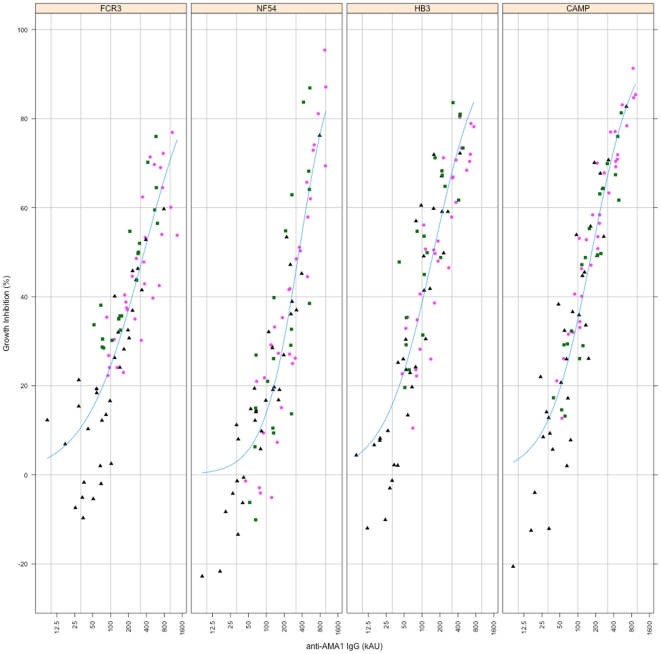
Relationship between ELISA antibody titre and *in vitro* parasite growth inhibition. Association of antibody levels with *in vitro* antibody functionality for three of the four immunisation groups (Gps 2, 3 and 4) is shown for parasite strains whose AMA1 allelic antigens were available for antibody measurement. In order to obtain an optimal estimate of the association, growth inhibition data at all four antibody concentrations tested (6.0, 3.0, 1.5 and 0.75 mg/ml) for each sample were included. Plots are based on a four-parameter logistic function, and each symbol/colour represents individual rabbits in the same immunisation group.

Since the proportions of strain-specific and cross-reactive antibodies as measured by competition ELISA were constant across immunisation groups, the lower GIA activity of the Montanide IMS group may be explained by the lower levels of the relevant cross-reactive antibodies measured in ELISA. Figure 6 shows that antibody levels against the relevant *Pf*AMA1 alleles correlate with the extent of *in vitro* inhibition of parasite strains that express those alleles. Thus the effect of adjuvant on the functionality of elicited antibodies is mainly on the quantity of cross-strain antibodies and not the quality of antibodies.

## Discussion

Antibodies to epitopes that are shared between different *Pf*AMA1 alleles (cross-reactive/conserved) have been shown to be relevant for broad strain parasite inhibition *in vitro* [16], [26], [27], and may be potentially important in the field [28]. We have previously shown that immunisation with mixed *Pf*AMA1 alleles induces a high proportion of such antibodies [15], and this represents an important strategy for dealing with allelic polymorphism in key antigenic vaccine targets like AMA1. The number of allelic variants of the antigen that are needed for a universally functional vaccine however remains a subject of discussion. This number will be dependent on the underlying mechanism of antibody production upon immunisation with a mixture of slightly different immunogens. Our current working hypothesis is that such a formulation will have strain-specific epitopes diluted out and this will preferentially enhance the presentation of shared epitopes to the relevant antibody-producing effector systems *in vivo*, thereby increasing the breadth of antibody response. Our Diversity-Covering approach is premised on this hypothesis, and in designing the DiCo proteins, polymorphic amino acids of high frequency (> 10%) in naturally occurring *Pf*AMA1 sequences have been incorporated two or three times [18]. Thus the DiCo proteins intrinsically have another level of coverage of polymorphism, and are expected to further increase the breadth of antibody response. This study compared the proportions of cross-strain antibodies elicited in separate rabbit immunisations with three and seven antigens, and also assessed the adjuvant effect on antigen-binding specificity of elicited antibodies.

Data from competition ELISA on plates coated with any one of five AMA1 antigens (DiCo 1, DiCo 2, DiCo 3, FVO or 3D7) showed that the binding specificities of antibodies induced in the seven-antigen immunisation were not distinguishable from those induced in the three-antigen immunisation (Gps 1 & 2 of Table 1 and Table 2). Thus the addition of four extra antigens to the three in the DiCo mix vaccine did not lead to a detectable increase in the proportion of antibodies against cross-reactive epitopes. Antibodies induced against a mixture of three *Pf*AMA1 alleles have been previously shown to exhibit greater cross-reactivity than antibodies against a single allele [15]. The current data thus suggests a plateau effect and a practical limit on the number of alleles that are needed to elicit the broadest antibody response possible.

Comparison of antibody depletion patterns of the four *Pf*AMA1 competitor antigens (FVO, HB3, 3D7 and CAMP) showed that the multi-allele vaccines least covered the 3D7 and CAMP AMA1 alleles, while HB3 and FVO AMA1 had reasonably better coverage (Tables 1 & 2). This was more so when assays were performed on DiCo 2 –coated (Table 1) or FVO AMA1-coated (Table 2) plates, and the observation was irrespective of the number of immunising alleles and the adjuvant used. Thus a fraction of antibodies elicited by multi-allele vaccine formulations is strain-specific. Alignment of the amino acid sequences of the three DiCo proteins alongside those of natural *Pf*AMA1 alleles (Figure 2) shows that apart from the cleavage site mutation (position K376R), there are five amino acids at polymorphic positions within the 3D7 AMA1 sequence (N34, R39, Y175, E197, P330) that are not present in any of the DiCo proteins, while all polymorphic amino acids in FVO AMA1 are present in at least one of the three DiCo proteins. Of the five unique amino acids in 3D7 AMA1, two in the prodomain (N34 and R39) do not have immunological significance since the *Pf*AMA1 prodomain is not present on the merozoite at the time of erythrocyte invasion [29], [30]. The glutamic acid residue at the heptamorphic hot-spot position 197 [31] forms part of a cluster of inhibitory epitope residues within domain I of the protein [26], and occurs in only 8% of 1294 *Pf*AMA1 sequences available (Remarque, unpublished data). The two other residues (Y175 and P330) are located in other clusters that are also deemed important in antigenic escape [26]. Although the seven-antigen vaccine includes 3D7 AMA1, there is likely an over-representation of antibody epitopes that are not found within 3D7 AMA1. Lower levels of antibodies will thus be elicited against the 3D7 AMA1-type epitopes, and this fits our working hypothesis. CAMP and HB3 AMA1 sequences have two (K228 and H407) and three (N34, R39 and K121) polymorphic amino acids, respectively, that are not covered in the DiCo sequences (Figure 2). Residue K228 is likely to occur within an important domain I cluster [26], while residue H407 is in domain II and has not as yet been described as part of an important escape cluster. Antibody epitopes that include these polymorphic amino acids may therefore be relevant for antigenic escape. Residue K121 occurs within domain I and has not yet been described as part of an escape cluster. Residues N34 and R39 occur within the prodomain and are immunologically not relevant.

Amino acids at positions 175 and 228 are in close proximity to the conserved tyrosine residue at position 251 within the hydrophobic trough of AMA1 [32]. This tyrosine residue is believed to be important for interaction with other invasion-associated proteins [32]. Residues at positions 330 and 407 cluster away from the hydrophobic trough and residue 121 occurs on the more conserved face of the molecule. Some of these polymorphic residues have been predicted to belong to discontinuous B-cell epitopes within AMA1 (DiscoTope method, referenced in [33]).

Functional antibody assay data supports the observations made with competition ELISAs. Antibodies from DiCo mix immunisation groups (Gp 2, Gp 3 and Gp 4) showed consistent levels of inhibition of five out of six parasite strains tested, with the L32 strain being an exception (Figure 4). In addition, anti-DiCo mix antibodies elicited with two of the adjuvants (CoVaccine HT™ and Montanide ISA 51) consistently showed the greatest inhibition of all strains. These data collectively indicate an improved breath of antibody response compared to antibodies from a single allele or two-allele immunisation [15], [16]. The data shows that strain-specific antibodies, despite being present as determined by competition ELISA, did not have any significant functional effects on parasites *in vitro*. Since strain-specific anti-AMA1 antibodies are highly inhibitory against homologous strains [9], [13], [34], it can be concluded that the multi-allele vaccines induced only very low titres of these antibodies, confirming our working hypothesis.

The functional activity of the antibody pool from the seven-antigen immunisation group compared favourably with a similar pool made from all eight sera in the DiCo mix CoVaccine HT™ immunisation group (Figure 3). Thus the seven-antigen vaccine does not have significant functional benefits over the three-antigen DiCo mix vaccine. Though inhibition of one of the parasite strains tested (HB3) was significantly higher for the seven-antigen immunisation pooled antibodies compared to the three-antigen pooled immunisation antibodies, this singular observation may not have biological significance.

The lower *in vitro* inhibition of L32 parasites is likely due to the extent of coverage of polymorphism of the L32 AMA1 sequence by the DiCo mix vaccine. Apart from mutations at the cleavage (K376R) and N-glycosylation (N162K, T288V, S373D, N422D, S423K and N499Q) sites, there are seven polymorphic residues (R197, D207, I224, N244, P330, R395 and T498) that are not present in any of the three DiCo proteins (Figure 5). Residues at positions 197, 207, 224 and 244 have already been identified within or in proximity of important antigenic escape clusters [26], and it is imaginable that these, in addition to any functional epitope(s) that include residues P330, R395 and T498, will contribute to significant levels of strain-specific antibodies to L32 parasites.

Taken together, the data largely suggests parasite inhibition through recognition of shared *Pf*AMA1 allele epitopes in these diverse parasites. These functional epitopes are likely to occur in AMA1 from many other parasite strains, and antibodies to such epitopes may play a key role in naturally acquired immunity to malaria [28]. Though partial, clinical immunity is acquired over time after repeated exposure to diverse parasite strains [35]–[38]. We therefore hypothesize that the antibody component of acquired immunity in semi-immune adults would be mainly to cross-strain epitopes, in contrast to the alternative model of a time-dependent acquisition of a diverse repertoire of strain-specific antibodies after repeated exposure to different parasite strains [28].

The choice of adjuvant for vaccine formulation has been shown to influence the quality and specificity of the elicited cellular and humoral responses [20], [39], [40]. In this study, adjuvant choice determined the levels of elicited antibodies and thus the extent of *in vitro* parasite inhibition observed (Figures 1 and 4). This however did not influence antigen specificity as antibodies induced in all four immunisation groups had near similar specificity profiles (Tables 1 and 2). This observation may be peculiar to DiCo mix as a vaccine candidate since the three DiCo proteins were designed to intrinsically cover polymorphism within natural *Pf*AMA1 sequences, and any specificity effects of the different adjuvants may have been masked by this property of the vaccine candidate. High levels of antibody of the right quality are required to counter parasite growth in both *in vitro* and *in vivo* systems [15], [41]. Thus the choice of adjuvant for a DiCo mix vaccine, based on the current data, may have little or no effect on the induction of the relevant cross-strain antibodies. This is important for this vaccination strategy since adjuvant influence on antibody specificity would introduce another level of complexity in achieving the ultimate functional cross-strain antibodies.

In summary, we show that the four extra *Pf*AMA1 alleles, when added to DiCo mix in a seven-antigen formulation, do not broaden the antigen specificity of elicited antibodies further, and that the three DiCo antigens may on their own be sufficient to cover *Pf*AMA1 polymorphism. The DiCo mix vaccine elicits functional broad-strain antibody responses directed mostly to shared epitopes. Though up to three relevant polymorphic amino acids in AMA1 sequences from FCR3, HB3, CAMP, NF54 and 7G8 parasite strains were not covered by the DiCo antigens, the consistent level of inhibition of these strains by anti-DiCo mix antibodies suggest that 100% coverage of polymorphic residues may not be necessarily required for a universal vaccine. The lower inhibition of L32 parasites, which have as many as seven potentially important polymorphic residues missing from the DiCo sequences however imply that the DiCo approach would need to be constantly reviewed, just as is the case for seasonal influenza vaccines. This is necessary to account for increases in the frequency of polymorphic amino acids that were low (<10%) at the time of DiCo design, and indeed for new polymorphic positions that are likely to evolve in the field. We finally demonstrate that different adjuvants elicit anti-DiCo mix antibodies with similar antigen specificities, although the absolute levels of elicited antibodies differ. This enhances the feasibility of developing an effective multi-allele AMA1-based vaccine with reduced formulation complexity and warrants further development of multi-allele immunisation strategies. We also highlight a potentially important role for cross-strain antibodies in naturally acquired immunity to malaria, and the need for novel adjuvants with improved antibody potentiation properties and safety for use in AMA1-based subunit vaccine development.
